# Comparison of mucin-1 in human breast cancer and canine mammary gland tumor: a review study

**DOI:** 10.1186/s12935-021-02398-6

**Published:** 2022-01-09

**Authors:** Rana Vafaei, Mitra Samadi, Aysooda Hosseinzadeh, Khadijeh Barzaman, MohammadReza Esmailinejad, Zohreh Khaki, Leila Farahmand

**Affiliations:** 1 Recombinant Proteins Department, Breast Cancer Research Center, Motamed Cancer Institute, ACECR, No.146, South Gandi Ave, Vanak Sq, Tehran, Iran; 2 Department of Clinical Pathology, Faculty of Veterinary Medicine, University of Tehran, Tehran, Iran; 3 Department of Immunology, School of Medicine, Iran University of Medical Sciences, Tehran, Iran; 4 Department of Surgery and Radiology, Faculty of Veterinary Medicine, University of Tehran, Tehran, Iran

**Keywords:** Mucin-1, Canine mammary tumor, Preclinical trials, Immunotherapy

## Abstract

Mucin-1 (MUC-1) is a transmembrane glycoprotein, which bears many similarities between dogs and humans. Since the existence of animal models is essential to understand the significant factors involved in breast cancer mechanisms, canine mammary tumors (CMTs) could be used as a spontaneously occurring tumor model for human studies. Accordingly, this review assessed the comparison of canine and human MUC-1 based on their diagnostic and therapeutic aspects and showed how comparative oncology approaches could provide insights into translating pre-clinical trials from human to veterinary oncology and vice versa which could benefit both humans and dogs.

## Background

The increasing demand in cancer research for more suitable immune-competent animal models, which develop cancer spontaneously, raised companion animals with naturally occurring tumors as a valuable resource [1–4]. Although comparative oncology, as a method for accelerating the development of drugs by introducing animal patients, is currently underused, it should continue to make a great opportunity to investigate many aspects of human cancers, from physiology to treatment [2, 5]. Canine mammary tumors (CMTs) are the most common malignancies in female dogs with a high mortality rate (which has been broadly characterized for genetic defects), bearing many common characteristics with human breast cancer (HBC) [6, 7]. Clinical and molecular similarities have been identified between HBC and CMT [3, 4, 7, 8]. Intact female dogs have a greater incidence of mammary tumors (84%), of which 78.2% lead to malignant forms; this may be due to the late submission of samples for histological examination [4]. CMT and HBC are similar in many ways, including environmental, biological, epidemiological, clinical, genetic, and pathological elements [9–11]. For instance, there is a great similarity of molecular and histological heterogeneity between CMT and HBC [9]. Moreover, the similarity between human and canine genome organizations are more than human and mouse [12], which proves that dog could be a better model for human cancer studies. Another notable benefit of using CMT for HBC research is that this cancer is the most common tumor in female dogs, with an incidence rate more than that seen in the human population [7]. Due to the lack of human tissue samples and the presence of ethical limitations in the research area, there is an urgent demand to find in vivo and in vitro alternative models for HBC [13]. Although cell lines are widely used in preclinical and in vitro studies, many believe that they are not a real model of cancer cells [13]. For in vivo studies, xenograft models and engineered dogs, cats, and mice are used [13]. Among them, dogs can be considered as an optimum animal model [13]. The reason is that many markers, which play a critical role in HBC, have the same role in canine tumorigenesis, such as the mucin family [13, 14]. Mucins are large, highly glycosylated proteins expressed by various secretory epithelial cells [14]. MUC-1 is also known as the cluster of differentiation 227 (CD-227), cancer antigen 15–3 (CA15-3), and Krebs von den Lungen-6 (KL-6). As the most thoroughly studied tumor-associated antigen (TAA), it is a type 1 transmembrane glycoprotein, which has a high molecular weight [15]. O-glycoprotein mucin is expressed at low levels on the apical borders of normal secretory epithelial cells [16]; it has a linearly extended extracellular domain, most of which contains tandem repeat sequences [17]. Same as other mucins, this glycoprotein is involved in protecting epithelial barrier, cellular adhesion, and lubrication, among other functions [18]. The identification of specific and sensitive molecular biomarkers involved in breast cancer has a consequential clinical significance; hence, MUC-1 has been broadly investigated for the treatment of HBC [19]. Overexpression and aberrantly glycosylation of this glycoprotein is related to the tumor invasion in human carcinomas and results in poor prognosis [20]. Also increasing concentrations of mucin-type glycoproteins in serum are due to increased tumor invasiveness in human [21]. MUC-1 expression is also known to be related to invasiveness and metastasis in CMT and has prognostic value [8, 20]. Thus, MUC-1 has become an interesting subject in the diagnosis and treatment of CMT and HBC [4, 14, 20, 22–24]. Many scientists believe that CMT is a perfect model for HBC investigations [9, 10]. CMT can be used for the evaluation of immunotherapy and other therapeutic applications [10]. Accordingly, this review drew a comparison between the MUC-1 roles in HBC and CMTs as a model for human studies.

## Gene and protein structure of mucin-1

The human MUC-1 gene of 4–7 kb length consists of seven exons, which can be alternatively spliced to make transcripts from 3.7 to 6.4 kb. This gene encodes a single polypeptide chain containing three different domains, i.e., a short C-terminal cytoplasmic domain (CD), short transmembrane domain (TD), and large N-terminal extracellular domain (ECTO) [25]. Tyrosine-phosphorylated CD is involved in signal transduction [26]. ECTO is made of a variable number (30–100) of 20 amino acids (GVTSAPDTRPAPGSTAPPAH), called variable number of tandem repeats (VNTRs) [18]. VNTR amino acid sequences can differ in various cancer cell lines as a result of the highly polymorphic character of this region. Dissimilar to the human MUC-1 sequence, mouse MUC-1 has 16 amino acid repeating tandems and bears only 34% sequence identity with its counterpart in humans [27]. An autocatalytic process in the endoplasmic reticulum cleaves this single polypeptide into two subunits as follows: (i) the MUC-1 N-terminal subunit (MUC-1 N-ter, MUC-1-N) that contains VNTRs and weighs more than 250 kDa, and (ii) MUC-1 C-terminal subunit (MUC-1 C-ter, MUC-1-C) that includes 58 amino acids of ECTO, whole 28 amino acids of TD, and whole 72 amino acids of CD, all weighing 23 kDa [28]. MUC-1-N is tethered to the cell surface by dimerization with MUC-1-C [28]. A transmembrane cleavage product of MUC-1, known as MUC-1*, which is the predominant form of this glycoprotein on cancer cells [29], functions as a growth factor receptor on tumor cells [29]. This cleavage of the full-length ECTO and formation of MUC-1* membrane receptor, appears to make binding to its ligand, NM23, possible [29]. CT and TD are highly conserved among species [25]. However, there are many transcriptional regulator elements in this region, which are not fully conserved between humans and mice [30]. Since the MUC-1-N subunit is imperfect with highly conserved variations [28], using a protein sequence homology analysis, it has been demonstrated that the CQCRRK sequence of MUC-1-C is fully conserved in dog and human proteins. Furthermore, dog MUC-1 CD shares a notable sequence homology of 83% with MUC-1 CD proteins of humans [31]. In HBC, known mutations of MUC-1 gene occurs as somatic changes within tumor genome [32]. On one hand, Carvajal-Agudelo et al. [33] found three single-nucleotide polymorphisms (SNPs) and two deletions (one in exon 7 and one in intron 6) in canine MUC-1. Furthermore, Carvajal-Agudelo et al. [33] found no significant correlation between MUC-1 expression and tumor grade or tumor type among CMTs; they found no correlation between MUC-1 and CMT incidence [33]. On the other hand, Manuali et al. showed that in CMT, MUC-1 expression is positively related to tumor grade; high MUC-1 in serum is found in grade II and III [24, 34]. MUC-1 overexpression is associated with poor prognosis in canine model [34].

## MUC-1 expression level and pattern of expression

In normal breast epithelial cells, MUC-1 is expressed at low levels just on the apical surfaces [19]. There is a different expression pattern of MUC-1 in both human and canine breast tissues, as well as in normal breast epithelial and tumor cells [14, 35]. Over the malignant transformation, the membrane expression of MUC-1 usually alters from apical to circumferential altogether with the loss of polarity of these epithelial cells, performing as anti-adhesive molecules, assisting the detachment of tumor cells, and thus increasing the metastatic and invasiveness of malignant cells [21]. The first study on MUC-1 expression in CMT was conducted in 2009 [14]. In this study, apical cellular localization of MUC-1 was found in normal canine mammary gland tissue but not in adjacent mammary tumors, and no significant association between the tumor histological type and the level of MUC-1 expression was found; this could be due to small sample size [14]. Meanwhile, in human studies, there was an association between high histopathological grading and MUC-1 over-expression [18, 36]. In 2012, a significant correlation was found between the MUC-1 expression level and the histopathological grade of tumor malignancy in human studies [24]. In a more recent study on canine MUC-1 expression, different expression patterns were detected among different CMT types; 92.8% were positive for cytoplasmic MUC-1 expression, 64.2% were positive for membrane MUC-1 expression, and 10.7% were positive for nuclear MUC-1 expression [20]. Furthermore, it has been proved that MUC-1 expression in primary tumors of dogs with metastasis to a regional lymph node is significantly higher than in tumors of dogs without lymph node metastasis [20]. Study in estrogen-receptor positive (ER+) human breast cancer have revealed that estrogen receptor α (ER α) resides in estrogen-responsive elements of the MUC1 promoter and triggers MUC1 transcription [37]. In CMT, the presence of estrogen receptor was correlated with pathological characteristics of the cancer, and the presence of ER seems to correlate with the degree of differentiation [38]. In particular, a lower expression of ERα was related to a worse prognosis, a larger size and skin ulceration [39].

## MUC-1 signaling pathway

**Fig. 1:**
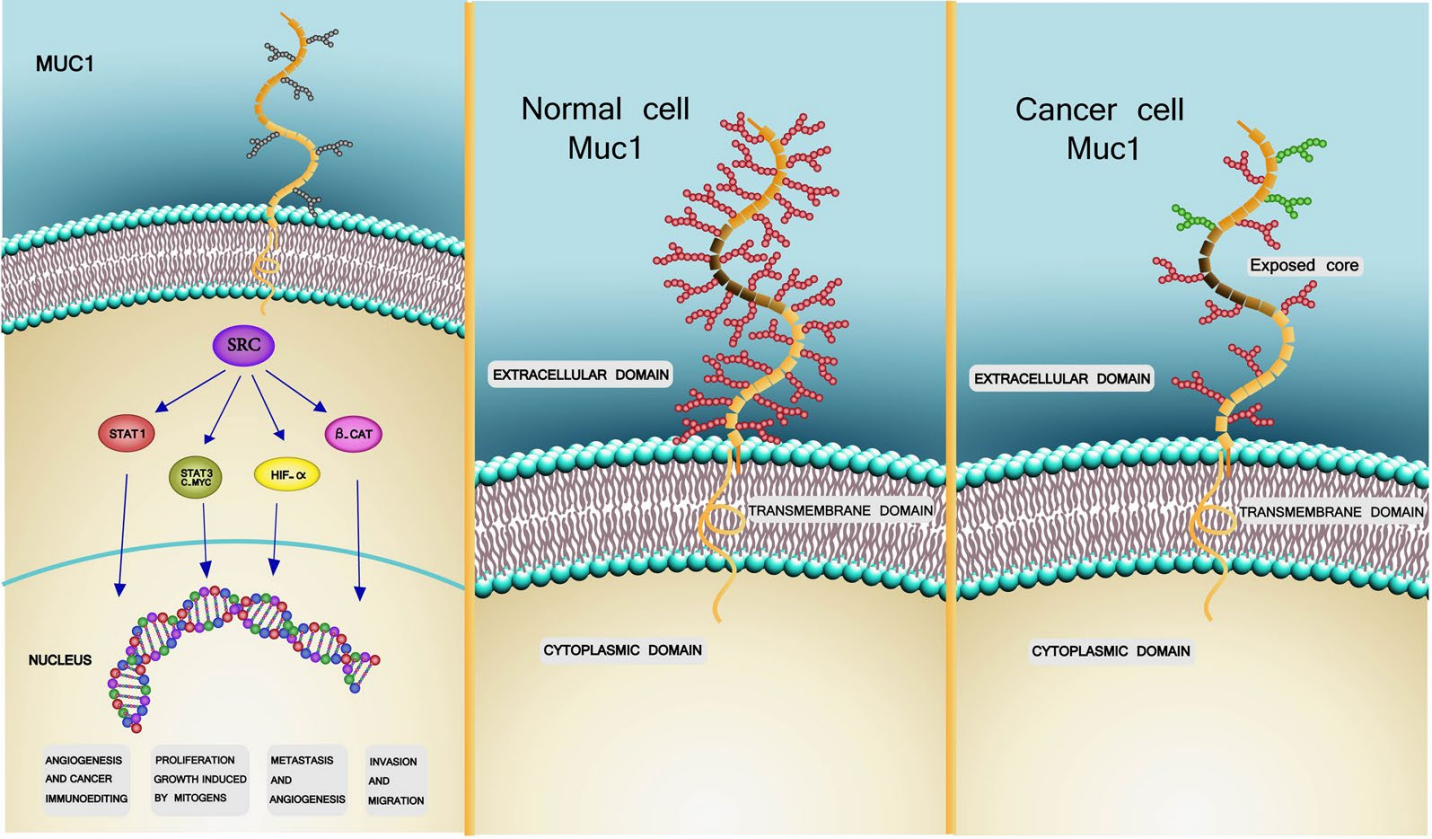
**A** MUC1 belongs to mucin family and its role in normal cells is protection of beneath cells from physical damages. In contrast, MUC1 play a different and complicated role in cancer cells. MUC1 cytoplasmic domain has crosstalk with other molecules like STAT1, HIFα and so on. These molecules are involved in growth, proliferation, angiogenesis, migration, invasion and metastasis. Because MUC1 has interaction with aforementioned molecules, it is a potential target in cancer therapy. **B** As illustrated above, MUC1 is hyper-glycosylated in normal cells. The tandem repeat, the black part including the APDTRRPAD amino acid sequence, is under the carbohydrate and consequently hided. **C** In cancer cells not only there is less carbohydrate density, but also new carbohydrates appear. Meanwhile, tandem repeat which was under carbohydrates before, becomes exposed. Thus, it can be concluded that MUC1 has distinct structure in normal and cancer cells

MUC-1 involves in apoptotic response in cancer cells and HBC, playing as a carcinogen in it [33]. The MUC-1-C transmembrane subunit interacts with receptor tyrosine kinases (RTKs), such as ErB2 and epidermal growth factor receptor (EGFR), at the cell surface and contributes to the activation of PI3K- Akt and mitogen-activated protein kinase (MEK)-extracellular signal-regulated kinase (ERK) downstream intracellular signaling pathways [31, 40]. MUC-1-C also contributes to activation of the Wnt/b-catenin cascade, signal transducer and activator of transcription (STAT), and nuclear factor-κB (NF-κB) RelA pathways by localizing to the nucleus (Fig. 1) [40].

As far as MUC-1-C is highly conserved among species [25] and acts as signal transduction to downstream effectors [40], we expect the same behavior in both canine and human signaling activation of this antigen. As evidence, a recent study on the CMT cell line (CMT-7364) showed that MUC-1 overexpression promoted the activation of the PI3K/Akt signaling cascade [8]. Zhao et al. showed in their research that MUC-1 interacted with AKt and by subsiding disulfiram effects on cancer cells in CMT, made them resistant to disulfiram [8]. By downregulation of MUC-1, AKt signaling was suppressed and this result established the crosstalk between MUC-1 and Akt [8]. In addition, they showed that with the activation of this pathway, cell migration and proliferation were also substantially promoted following its overexpression [8].

## CA15-3 detections in peripheral blood of dog and human

Circulating markers, such as glycoprotein substances, are measurable in blood and body fluids in both neoplastic and health conditions. They usually have higher levels of neoplasia and are known as tumor indicators and prognostic tools [4]. MUC-1-N truncates and sheds blood from breast cancer cell surfaces and is found in the serum of women with metastatic breast cancer at increased levels [40]. The soluble form of MUC-1 in peripheral blood is called CA15-3 [41], extremely expressing in human mammary malignancies, and is one of the best known and most broadly used serum tumor markers in women with breast cancer [24]. This marker also is used as a monitoring tool for response to treatment [42]. Furthermore, there is a correlation between the circulating CA15-3 level and histopathological grade of breast cancer [24]. Accumulating evidence suggests that this glycoprotein is a potential promising marker in canine medicine in addition to human medicine; therefore, here, we provided a summary of these pieces of evidence. For the first time, Clinton et al. [43] studied the diagnostic efficacy of dogs’ serum CA15-3 and found 63% diagnostic sensitivity and 80.64% specificity using the Centocor CA15-3 assay (Fujirebio Diagnostics, Centocor Inc), which is a solid-phase radioimmunoassay (RIA). They used the 115D8 murine monoclonal antibody (mAb; as the capture antibody), besides the I125 labeled DF3 murine mAb; thus, it was concluded that CA15-3 could be considered the best tumor antigen regarding diagnostic aid and monitoring agent [43]. Five years later, using IMMULITE appliance and human kits (Medical System S.P.A, Genoa), Marchesi et al. [44] measured CA15-3 in serum and plasma of clinically healthy dogs/non-neoplastic diseases and dogs with clear tumor lesions other than mammary tumors. They concluded that the determination of CA15-3 could not differentiate among any of their study groups [44]. In another study, they performed the measurements using ADVIA Centaur by the direct chemiluminescence method and human kits (Bayer Immuno 1 CA15-3) and found it applicable for the determination of this marker in canine mammary oncology diagnostics [4]. They reported that subjects with serum CA15-3 levels higher than 1 U/mL were found to have mammary neoplasia based on pathology assessments [4]. In a study by Campos et al. [42], a significant positive correlation between mammary neoplasm staging and this serum marker was observed. Also, in agreement with the previous study, the serum concentration of CA15-3 was significantly less in dogs with no evidence of mammary tumors compared to dogs with benign and malignant mammary masses. Three years later, they also found a significant increase in serum CA15-3 in dogs with regional lymph node metastatic mammary neoplasms compared to those without metastasis and healthy dogs. In this study, authors used commercial solid-phase, non-competitive ELISA immunoassay kits [20]. In the most recent study on this field, this marker was analyzed using canine-specific kits of Bioassay Technology Laboratory, and it was found to be higher in dogs with mammary cancer compared to controls (tumor-free dogs) with high sensitivity, supporting the aforementioned studies [45]. These findings reflect the fact that this tumor marker could not only be used in dogs as a model for human studies but also is a promising diagnostic tool in canine oncology.

## Immunogenicity of MUC-1

MUC-1 immunogenicity was examined in mice and human models [46]; the results showed that MUC-1 induced humoral and cellular immunity [47]. In the case of cellular immunity, cytotoxic lymphocyte (CTL) cells were isolated from BC patients, reacted to the MUC-1 tandem repeat [46, 48]. Also, there is a humoral response to MUC-1; some patients with benign tumors had anti-MUC-1 antibodies in their blood and showed a better prognosis [46]. Indeed, the results demonstrated that anti-MUC-1 immunoglobulin G (IgG) could bind to MUC-1 in some BC patients and mice models [46]. Numerous studies have been conducted to know which part of MUC-1 can induce immune response successfully. In this regard, the results have indicated that small epitopes of MUC-1 are highly immunogenic and targeted by mAbs [49]. Moreover, the CTL cells harvested from patients recognized the same epitope of MUC1, which was identified by mAbs [49, 50]. Cancerous MUC-1 has short-chain O-glycosylation with low density; this structure allows the specific amino acid sequence to be exposed to the cell surface [51, 52]. In this position, Tn and STn epitopes are accessible to the immune system [51]. This structure belongs to cancer cells uniquely and results in the discrimination of a mAb between normal and cancer cells [51].

On the other hand, autoantibodies are produced by humoral immunity against low levels of tumor-associated antigens as a very specific antibody response, supplying an early cancer detection tool [18, 22]. Anti-mucin immunoglobulins (i.e., MUC-1-IgG and MUC-1-IgM) are one of these circulating antibodies [18]. The complex of MUC-1 with these antibodies can also be found in the bloodstream [53]. When the MUC-1 level elevates in circulation, the anti-MUC-1 antibody titer decreases concurrently; this may be caused by their binding and formation of the antigen–antibody complex [53]. Anti-MUC-1 autoantibodies have been observed in both HBC and CMT [22] which proves that MUC-1 induces immune response in human and dog. Although these autoantibodies have been known as breast cancer biomarkers, the problem is that autoantibodies against aberrantly glycosylated MUC-1 are also present in healthy people and benign diseases, which makes it hard to differentiate between cancer and healthy titers [18]. Although a higher titer of circulatory anti-MUC-1 antibodies was found in humans with breast cancer [22], to the best of our knowledge, the first and only study on autoantibodies against MUC-1 in canines was recently conducted in 2018 [22]. To demonstrate the diagnostic potential of several autoantibodies (such as MUC-1) in CMT, they took advantage of a high-throughput Luminex technique [22]. Their results showed that the anti-MUC-1 autoantibody assay had the highest sensitivity and specificity (62.67% and 98%, respectively) among other anti-TAA antibodies, including triosephosphate isomerase (TPI), phosphoglycerate mutase 1 (PGAM1), manganese superoxide dismutase (MnSOD), and avian myelocytomatosis viral oncogene homolog (c-Myc), to detect mammary tumors in dogs [22]. According to the therapeutic, immunogenicity, oncogenicity, epitope numbers, and expression level, the National Cancer Institute (NCI) ranked MUC-1 as the second target for clinical goals [54]. Mucins can block CTL, natural killer (NK) cells, and neutrophil activity and consequently inhibit anti-tumor immune response [49]. MUC-1 inhibits the binding of immune cells to their target and hinders the immunotherapy effectiveness in cancers with epithelial cells originally [49]. The reason is that MUC-1 is a self-antigen, even in its specific structure on cancer cells tolerance, which does not allow the immune system to respond properly [47]. Hence, some techniques have been used to improve immune response to MUC-1, especially increasing the efficacy of vaccines [47]. For instance, some researchers have used tumor-specific carbohydrates (inducing an immune response), which may lead to less tolerance to these tumor-specific carbohydrates in patients [47].

## Canine immune response to mammary tumors

Studies show that immune subsets of human and canine are similar [10], furthermore, the immune response to mammary tumors shows many similarities in human and dogs. According to several research results, not only the composition of immune cells in tumor microenvironment in HBC and CMT is alike, but also results demonstrate a parallel expression pattern of immune molecules in both tumors [10]. In cellular level, CMT cells like HBC cells show high number of neutrophil cells [55], TAMs [10, 56], and CD4 + [12, 55, 56] cells which are associated with metastasis [55, 56]. Furthermore, high number of plasma cells, macrophages, CD8+ are negatively related to metastasis [55]. Like HBC, in CMT M2 macrophages and CD3+ T cells play a role in angiogenesis, in both HBC and CMT, M2 macrophages secrete VEGF [56]. In molecular level, the expression of programmed death-ligand 1 (PDL1) and PDL2 two co-inhibitory molecule- have been examine in CMT cells. PDL2 expression is reduced in CMT metastatic tumors, similar phenomenon happens for PDL1 expression in metastatic HBC [10]. Gal9 boosts anti-tumor immunity mediated Th1 cells in HBC and CMT [10]. Gal9 is a prognostic marker in both models and in human, gal9 expression is negatively related to distant metastasis, moreover, in CMT, gal9 expression in reduced in tumor cells [10]. IL-10 is a robust anti-inflammatory cytokine and is secreted by immune cells like T-reg cell and cancer cells, its expression is upregulated in HBC and CMT [10].

## The role of MUC-1 in cancer immunotherapy

Immunotherapy is one of the newest strategies used in BC. Since there are some specific problems and complexity with other strategies of BC therapy (such as chemotherapy, radiotherapy, and surgery), the need for a new strategy made researchers propose immunotherapy [57, 58]. Different immunotherapy methods, including mAb, vaccine, chimeric antigen receptor (CAR) T cells, have been investigated in BC. For example, trastuzumab is the most known agent that has been utilized in HER2 + BC patients and improved prognosis in these patients [59].

### Immunotherapy in canine tumors

There are few examples of immunotherapy approaches which have been used in canine cancers like melanoma and lymphoma. Some of these approaches are used for treatment of canine cancers, while others have been under investigation in clinical phases. For example, anti-CD20 mAb is used for treatment of lymphoma in dogs [12]. Moreover, dogs with metastatic melanoma have been treated by Indoleamine 2,3-dioxygenase (IDO) inhibitors in combination with radiotherapy [12]. On the other hand, cDNA vaccine- targeting tyrosinase- has been investigated in phase II and III clinical trial for oral melanoma [12]. Different settings of HBC immunotherapy have been under investigation, which target MUC-1. These settings include vaccine, mAb, CAR T cells, and combination immunotherapy. The following is a survey of these settings and their results. By reading the following survey, we should consider MUC-1 as a potent therapeutic target not only for HBC but also for CMT.

### Anti-MUC-1 vaccine

Researchers have examined the effects of the MUC-1 subunit vaccine [keyhole limpet hemocyanin (KLH) and DETOX as an adjuvant] in BC patients [60]. The results of this study showed that this vaccine exploited CTLs restricted MHC-I response [60]. In different settings, the MUC-1 DNA vaccine and GM-CSF were evaluated in a murine model of BC [60]. The results demonstrated that this vaccine could reject tumors in mice [60]. The MUC-1 glycopeptide vaccine is another type of the MUC-1 vaccine that has been investigated. Tetanus toxin and sialyl-Tn epitope of MUC-1 were injected into mice bearing BC and decreased tumor growth in them [60]. A MUC-1/HER-2 chimer protein was examined in a BC mice model, and its results showed that humoral and cellular immune response was induced in these animals [54]. In this study, mice produced anti-MUC-1/HER-2 IgG and T helper 1 response [54]. This vaccine increased cancer cells’ necrosis and reduced lung metastasis [54]. The anti-STn vaccine could induce IgG and IgM production in BC patients [49].

MUC-1 and toll-like receptor 7 (TLR-7) peptide in a murine model of BC were tested, and this vaccine induced CTL response and antibody-dependent cellular cytotoxicity (ADCC) and improved humoral immunity against MUC-1 + cancer cells [61]. This vaccine might inhibit tumor growth and recurrence and showed significant cytotoxicity in vivo [61]. A virus-like particle containing MUC-1 and survivin peptide was evaluated in a triple-negative breast cancer (TNBC) mice model, which specifically induced cytotoxicity and CTL response [62]. The conserved MUC-1 peptide and dendritic cells (DCs) induced immune response against tumor cells [51]. In a murine BC model, the MUC-1 liposome vaccine and interleukin 2 (IL-2) were investigated; it could induce interferon gamma (IFN-γ) secreting T cell, which target MUC-1 [46]. In a clinic, in order to overcome self-tolerance to MUC-1, in a study, HLA-A0201-MUC-1 and allogenic DC were tested in BC patients, which could create CTL response in them [47]. In MUC-1 vaccines, different adjuvants have been investigated to boost immune response. For instance, MUC-1 and tetanus toxin elicit a stronger immune response compared to the synthetic MUC-1 peptide alone [63]. In TNBC patients, the MUC-1 peptide and poly polyinosinic:polycytidylic acid (ICLC; an immune stimulator) were under investigation in clinical phase [64]. Tan et al. [65], in phase I clinical trial, examined an adenoviral vector vaccine, which carried a MUC-1/CD-40 peptide. In clinical settings, researchers have investigated the efficacy of autologous DC, which carried MUC-1 complementary DNA (cDNA) in advanced BC patients [60]. The patients showed T CD8 + response, specifically bound to MUC-1 and secreted IFN-γ [60]. In a clinical trial phase II study, sialyl-STn in conjugation with KLH was tested in BC patients, which showed no efficacy [60].

### Monoclonal antibody

There is a long list of mAb and antibody fragments, which have been applied in BC [59]. These agents targeted different antigens (such as HER2, VEGF, and so on) and improved the overall survival and quality of life of BC patients [59]. Hence, it can be assumed that mAb and antibody fragments are the most successful immunotherapeutic agents in BC. Also, mAbs against MUC-1 can be used as diagnostic and therapeutic agents in BC [49, 51]. Most of the anti-MUC-1 mAbs react to cancerous tissue with high affinity and show low affinity to normal tissue [49]. Hence, it can be concluded that these mAbs target cancer cells specifically. There is a murine mAb that recognizes Tn and STn in MUC-1 [51]. Another mAb, SM-3, is an anti-MUC-1 mAb, which binds to MUC-1 specifically [66]. SM-3 and CTL cells recognize the same epitope of MUC-1 [66]. The clinical outcomes of anti-MUC-1 mAbs produced in mice have not been promising [51]. This failure was due to the source of these antibodies. On the other hand, it is possible that a humanized antibody could promote ADCC successfully [51]. Zhou et al. [52] produced a TAB004 anti-MUC-1 mAb, which did not react to normal tissues and specifically bound to cancer cells. TAB004 targeted 95% of all MUC-1+ malignant tissue, including TNBC samples [52]. Panchamoorthy et al. [67] generated an anti-MUC-1-C mAb and showed that it could bind to BC cell lines and induced cytotoxicity responses; it also showed the anti-tumor activity in mice bearing BC xenograft. They suggested that their mAb can be used as a drug conjugated mAb [67].

### Application of CAR T cells

CAR T cells are T cells engineered TCR and are one of the immunotherapy strategies [58]. Because T cells play a central role in the immune system, using them in cancer therapy is an interesting research area. Therefore, several groups worked on CAR T cells [51]; for example, in one study, anti-MUC-1 specific CAR T cells were produced and showed significant anti-tumor activity and cytotoxicity in TNBC in vivo and in vitro [52].

### Combination immunotherapy

Using single-agent immunotherapy achieved success in BC; however, problems such as resistance appear [58]. To target cancer more effectively, combining different immunotherapy agents with distinct targets could be more useful [58]. In BC targeting, PD-1 and CTLA-4 by mAbs were approved by the US Food and Drug Administration [57, 58]. Considering the fact that MUC-1 has been examined in immunotherapy lately, MUC-1 has been introduced to the combination immunotherapy field in BC. There is a combination of the MUC-1 messenger RNA (mRNA) vaccine and anti-CTLA-4 targeting mAb, which was tested in the TNBC animal model [68]. In this study, this combination strategy prevented tumor growth in tested mice.

## Conclusions and future perspectives

Nowadays, using animals with spontaneously occurring tumors has received a great deal of attention in human studies as a proper cancer model and also to “translating clinical trials from human to veterinary oncology and back” [5]. The unique features of CMT, such as occurring spontaneously, size similarities, identical clinical stages, rapid growth, etc., have made this tumor an interesting choice for comparison studies. Another fascinating aspect of utilizing CMT in human studies is the potential to exhibit the same molecular characterizations in both species. In this aspect, MUC-1 is one the most thoroughly studied TAAs in human research, though narrowly assessed in canine and comparison investigations. Thus, in order to find a more promising potential of MUC-1 in diagnostic and therapeutic facets of comparison cancer research, here, we summarized the current knowledge about the different aspects of this biomarker in human and canine views.

Gene and protein structure similarities, as well as the level and pattern of expression, along with common signaling pathways, make canine MUC-1 a proper candidate for human studies. However, in canine oncology, the role of MUC-1 in tumors is less investigated and it is undeniable that there are also many challenges and risks that are preventing the practical using of this model. Lack of potent infrastructures which provide coordination among veterinary hospitals, researchers, oncologists and pharmaceutical companies are important points which have barricaded the potential application of such a model [7]. Identification of breed specific risk factors of CMT is also a necessity [7]. One recommendation is to conduct more studies on the subject of canine MUC-1 together with getting through the comparison research of MUC-1.

## Data Availability

Not applicable.

## Abbreviations

MUC-1: Mucin-1
CMT: Canine mammary tumor
HBC: Human breast cancer
SNPs: Single-nucleotide polymorphisms
CD: Cluster of differentiation
CA: Cancer antigen
KL: Krebs von den Lungen
CD: Cytoplasmic domain
TD: Transmembrane domain
VNTR: Variable number of tandem repeats
RTKs: Receptor tyrosine kinases
EGFR: Epidermal growth factor receptor
MEK: Mitogen-activated protein kinase
ERK: Extracellular signal-regulated kinase
STAT: Signal transducer and activator of transcription
ER+: Estrogen-receptor positive
MAB: Monoclonal antibody
CTL: Cytotoxic lymphocyte
TAA: Tumor-associated antigen
IgG: Immunoglobulin G
TPI: Triosephosphate isomerase
PGAM: Phosphoglycerate mutase
MnSOD: Manganese superoxide dismutase
Myc: Myelocytomatosis
NCI: National Cancer Institute
NK: Natural killer
PDL: Programmed death-ligand
CAR: Chimeric antigen receptor
IDO: Indoleamine 2,3-dioxygenase
TLR: Toll-like receptor
ADCC: Antibody-dependent cellular cytotoxicity
TNBC: Triple-negative breast cancer
DC: Dendritic cells
IFN: Interferon gamma
cDNA: Complementary DNA
FDA: Food and Drug Administration
mRNA: Messenger RNA
